# A Cluster of *Candida auris* Blood Stream Infections in a Tertiary Care Hospital in Oman from 2016 to 2019

**DOI:** 10.3390/antibiotics9100638

**Published:** 2020-09-24

**Authors:** Jalila Mohsin, Sanjeewani Weerakoon, Sarah Ahmed, Ynze Puts, Zainab Al Balushi, Jacques F. Meis, Abdullah M.S. Al-Hatmi

**Affiliations:** 1 Department of Microbiology, Royal Hospital, Ministry of Health, 111 Muscat, Oman; jalila76.mohsin@gmail.com (J.M.); sanjee707w@gmail.com (S.W.); zezo_albalushe@hotmail.com (Z.A.B.); 2 Foundation Atlas of Clinical Fungi, 1214 GP Hilversum, The Netherlands; arah.AhmedIbrahim@radboudumc.nl; 3 Centre of Expertise in Mycology Radboud University Medical Centre/Canisius Wilhelmina Hospital, 6532 SZ Nijmegen, The Netherlands; jacques.meis@gmail.com; 4 Department of Medical Microbiology and Infectious Diseases, Canisius Wilhelmina Hospital, 6532 SZ Nijmegen, The Netherlands; Y.Puts@cwz.nl; 5 Bioprocess Engineering and Biotechnology Graduate Program, Federal University of Paraná, 80010 Curitiba, Brazil; 6 Ministry of Health, Directorate General of Health Services, 514 Ibri, Oman

**Keywords:** *Candida auris*, candidemia, infection, risk factors, genotyping, Royal Hospital, Oman

## Abstract

(1) Background: *Candida auris* has been reported as emerging yeast pathogen that can cause invasive bloodstream infections in healthcare settings. It is associated with high mortality rates and resistance to multiple classes of antifungal drugs and is difficult to identify with standard laboratory methods. (2) Methods: We conducted a retrospective review of epidemiological, clinical, and microbiological records for 23 *C. auris* fungemia cases at the Royal Hospital, a tertiary care facility in Oman, between 2016 and 2018. Demographic data, risk factors associated with mortality, microbiology investigation and treatment regimens are described. Yeasts were identified by MALDI-TOF. (3) Results: We identified 23 patients with *C. auris* fungemia. All positive samples from patients were confirmed as *C. auris* using MALDI-TOF, and ITS-rDNA sequencing. Microsatellite genotyping showed that the Omani isolates belong to the South Asian clade I. The majority of patients had multiple underlying illnesses and other risk factors that have been associated with fungemia. All isolates were non-susceptible to fluconazole. Isolates from all patients were sensitive to echinocandins and these were used as first line therapy. (4) Conclusions: *Candida auris* affects adults and children with a variety of risk factors including central venous catheters and overuse of antibiotics. Infections occur in both immunocompromised and immunocompetent individuals. Mortality was high in this series, and the organism can be transmitted in healthcare settings. Programs for raising awareness in Oman hospitals are warranted. Caspofungin remains 1st line therapy as MICs are still low despite its wide use.

## 1. Introduction

Bloodstream fungal infections are a significant cause of mortality in immunocompromised patients, including AIDS, organ transplant recipients, hematology and oncology patients and patients requiring invasive therapies [1]. *Candida* spp. account for 70 to 80% of invasive bloodstream fungal infections, particularly in ICU patients, and they represent the fourth most common nosocomial bloodstream infection [2,3,4]. Although *C. albicans* is still the most commonly isolated species among *Candida*, recent worldwide studies showed that the epidemiological landscape of candidemia is shifting toward an increasing prevalence of non-albicans species, particularly *C. tropicalis*, *C. glabrata*, *C. parapsilosis* [5] and most recently *C. auris,* which has emerged globally causing up to 60% of candidemia cases [6,7]. In the United States, over 30% of cases of candidemia are caused by *C. glabrata*, and about 20% of cases caused by *C. parapsilosis* [5], whereas *C. parapsilosis* is mainly reported from Australia, Europe and Latin America [8]. Non-albicans species often have reduced susceptibilities or even intrinsic resistance to echinocandins or azoles, particularly fluconazole [9].

Ten years ago, *C. auris* was first discovered, and since then, it has quickly become a predominant infectious pathogen with increasing global prevalence [10,11]. *Candida auris* is currently one of the most common clinical fungal pathogens, causing nosocomial infections [7]. Especially ICU patients with medical devices such as central venous catheters (CVC) or urinary catheters, treatment with broad-spectrum antibiotics, long hospital stays, major surgery and immunosuppressive therapy are affected [1,12,13]. Because of higher drug-resistance rate, *C. auris* is more difficult to treat, requires longer hospitalization periods, and results in higher morbidity and mortality than other *Candida* species [14]. 

In 2017, the first cases of *C. auris* from Oman were reported [15,16], and in 2019, we reported an outbreak in the city of Sohar [12]. In this study, we report 23 cases of *C. auris* fungaemia diagnosed at a tertiary care center in Muscat, Oman. We reviewed demographic data, risk factors, treatment, and outcomes associated with *C. auris* fungemia at our institution during a 4-year period from 2016 through 2019. In addition, susceptibility testing and genotyping of *C. auris* isolates associated with fungemia were performed, changes in susceptibility patterns during the 4-year period were assessed, and the outcomes of infection were correlated with antifungal susceptibility values.

## 2. Results

### 2.1. Clinical Characteristics and Manifestations

**Figure 1:**
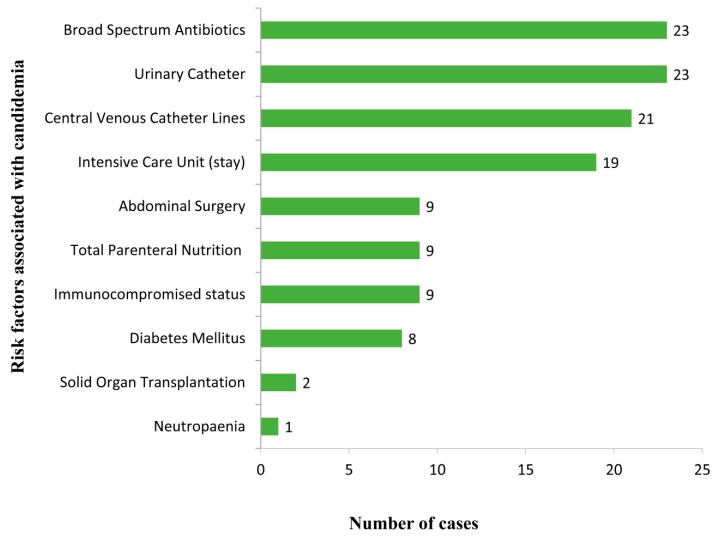
Risk factors for *Candida auris* infections in hospitalized patients (*n* = 23).

Between January 2016 and December 2019, 23 patients with fungemia due to *C. haemulonii* species complex and later confirmed by genetic and proteomic techniques as *C. auris* were identified. Complete medical records and the initial *C. auris* isolate were retrieved. Demographic data, clinical manifestation, risk factors, treatment, and outcome are shown in Figure 1. There were 21 adults and 2 pediatric patients with median age of 51 years and 60% were male. The majority of patients had multiple underlying illnesses and other risk factors that have been associated with fungemia. The most common underlying illness and predisposing factors were use of broad-spectrum antibiotics (100%), use of urinary catheters (100%), and use of CVC (91.3%). Patients stayed in an ICU (87%) with an average length of 35 days before fungemia was diagnosed (range from 7–120 days). One pediatric patient (4.3%) was neutropenic. Nine patients had diabetes mellitus and six patients had undergone major abdominal surgery, with four of them receiving total parenteral nutrition. The majority had positive blood cultures (82.6%). In our study, out of the 23 infected patients, 9 died (39.1%) and 14 (60.9%) recovered.

### 2.2. Microbiology

**Figure 2:**
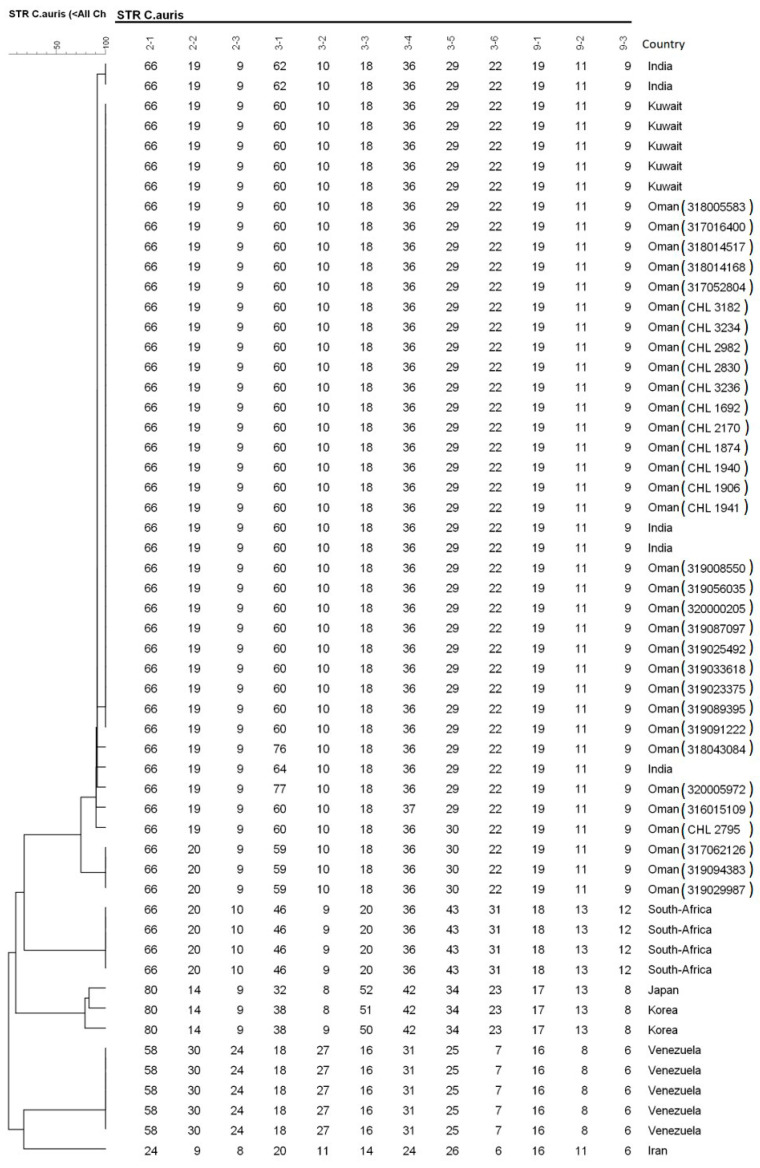
Short tandem repeat (STR) for *C. auris*, 12 STR targets with a repeat size of 2, 3 or 9 nucleotides. The isolates from Oman clustered with Indian and Kuwaiti isolates in the South Asian clade 1, while isolates from South Africa, Japan/Korea, Venezuela and Iran each clustered in four different clades.

All blood isolates were detected from automated blood cultures (BACTEC/BD) within 48–72 h of incubation. Yeast isolates grew well as white cream colored colonies at 37 °C and 42 °C aerobically on Sabouraud’s agar media. Identification of isolates was performed by different methods: phenotypic (BD Phoenix and API AUX 20C), proteomic (MALDI-TOF) and molecular using ITS-sequencing. The Phoenix Identification system identified all the isolates as *C. haemolunii*, whereas API AUX 20C provided several identifications, including *C. famata* (in majority), *Rhodotorula glutinis*, *C. haemolunii* and *C. glutinis*. MALDI-TOF and ITS-sequencing identified all isolates as *C. auris*. All isolates evaluated by short tandem repeat (STR) genotyping belonged to a single clade and clustered with Indian isolates in the South-Asia clade 1 (Figure 2), while isolates from South Africa, Japan/Korea, Venezuela, and Iran each clustered in the other four major *C. auris* clades. In vitro antifungal susceptibility testing showed elevated MICs for fluconazole and amphotericin B. Although the Clinical and Laboratory Standards Institute (CLSI) does not provide specific antifungal break points for *C. auris*, according to available data, our isolates showed resistant to fluconazole and variable susceptibility to voriconazole and amphotericin B. All isolates had wildtype echinocandin MICs (Table 1). 

### 2.3. Treatment and Outcome

All patients received antifungal treatment with echinocandins (caspofungin or anidulafungin according to the availability of the drug) with a minimum duration of 14 days. Some patients received combination treatment with amphotericin B later during treatment. In addition, CVCs were removed as source control measures and patients were kept in an isolation room with contact precautions.

## 3. Discussion

*Candida auris* is increasingly reported as a cause of fungemia and invasive candidiasis worldwide. In this study, we evaluated *C. auris* bloodstream infections which mainly occurred in immunocompetent patients (*n* = 18), and only five patients were immunocompromised. Our patients were exposed to long-term broad-spectrum antibiotics, to medical procedures including central venous and urinary catheters, major abdominal surgeries, and long-term stay in ICUs. In this study, clinical presentation was non-specific, and it was difficult to differentiate between other types of systemic infections in most cases. *Candida auris* infections are usually diagnosed by laboratory tests such as culture of blood or other body fluids. Most common symptoms are fever and chills that do not improve with antibiotic treatment. Risk factors are similar to previous studies [7,13,17,18,19,20]. Rudramurthy et al. [21] studied fungemia caused by *C. auris* including 74 patients from 19 ICUs in India. The authors reported that *C. auris* infection was associated mainly with longer ICU stay, excessive antifungal exposure and respiratory infection, but there was no association with the presence of CVC. Other potential risk factors such as smoking, high blood pressure and diabetes are generally associated with ICU admission, but it is unclear if they are specific for *C. auris* candidemia. Factors that have led to the emergence and rapid spread of *C. auris* in hospitals in Oman are not well established. We believe that one patient who came from India had a CVC already colonized with *C. auris* and might have spread the infection in the ICU. This patient went to India for treatment following a C5-6 level fracture, had been treated at an ICU in India, received multiple course of antibiotics for a hospital acquired pneumonia, and returned to Oman for further treatment. He was directly admitted to the Royal Hospital with a CVC in situ from which a blood culture became positive for yeasts. In addition, risk assessment for *C. auris* was not included in the institutional screening protocol during this period, and this might have caused evidence of an epidemiological link in our clinical setting to be missed. *Candida auris* has the ability to colonize skin, mucosal tissues, nasal cavities, dry and moist surfaces, floors, sinks and beds [22,23,24,25]. In the healthcare setting, it is believed that moist surfaces allow *C. auris* to colonize and spread in hospital environments more than dry surfaces. Similar observations were made with bacteria such as methicillin-resistant *Staphylococcus aureus* (MRSA) and carbapenem-resistant *Enterobacteriaceae* (CRE) [22]. In this study, only mycological resistance was investigated to see if the fungus is able to grow in the presence of antifungal drugs that would otherwise kill them or limit their growth in vitro, but clinical resistance was not investigated. All isolates were sensitive to all tested echinocandins (anidulafungin and micafungin) since they exhibited MICs of ≤4 mg/L at 24 h.

As reported previously and shown again in the current study, *C. auris* fungemia is seen more often in older adults (*n* = 21) than in children (*n* = 2) [12,26,27]. Apparently, male patients accounted for a higher proportion than females in this series from Oman. A similar observation was made in a meta-analysis of 742 *C. auris* isolates from 16 countries, mainly from India (*n* ≥ 243), USA (*n* ≥ 232) and UK (*n* ≥ 103) during the period 2013–2017. Most isolates were from males (64%) and mostly from blood (67%) [27,28,29]. The reasons behind this difference between sexes are not known yet in *C. auris* infections [29]. However, *C. auris* case differences between sexes might be country-specific and local health practices might also play a role in the higher male rates recorded per country.

The 30-day crude mortality rate of *C. auris* infection in general has been variable in different geographical regions and found to range between 0% and 72% [12,23,30]. In our study, out of the 23 infected patients 9 died (39.1%) and 14 (60.9%) recovered. Mortality in this report was within the international mortality record for *C. auris*. In comparison, a recent Kenyan study with 21 *C. auris* patients reported a crude mortality rate of 29% [31], whereas in another study from USA, 41 *C. auris* patients were included in a worldwide collection of *C. auris* isolates, and this study reported a crude mortality rate of 59% [32]. In contrast, the crude mortality rate (39.1%) is lower than our previously published outbreak, with a rate of 53.1% [12]. Few studies on *C. auris* candidemia have been conducted in the Middle East [33,34,35], and it was reported that the crude mortality rate was about 60% [33]. The high mortality rate could be due to several reasons such as the presence of comorbidities, diagnostic delays and the resistant nature of the pathogen [29]. Furthermore, in our study, neutropenia was observed only in one pediatric patient.

Identification is not easy with conventional diagnostic tools, and misidentification was reported since *C. auris* is closely related with *C. haemulonii*, *C. lusitaniae* and others. However, it is important to have a suspicion if an oval to elongated yeast without pseudohyphae-forming pink-to-beige colonies on chromogenic agar, and which is able to grow at 42 °C, is encountered [36]. MALDI-TOF MS and sequencing are advanced and perform well in identification of *C. auris,* but are relatively expensive techniques. Thus, there is critical necessity to develop cost-effective techniques for the detection of this emerging pathogen in less developed countries [37]. Many countries including Oman face difficulties in the identification of *C. auris,* and also the unavailability of antifungal susceptibility testing continues to be a challenge when managing patients with *C. auris*. *Candida auris* is currently separated into five geographically restricted clades (East-Asia, South-Asia, Africa, Iran and South-America) [38]. In the present study, we typed 55 isolates, originating from India, Kuwait, Oman, South-Africa, Japan, Korea, Venezuela and Iran with STR genotyping [39], which showed that the Omani isolates belong to the South Asian clade and had a clonal nature. STR genotyping also agreed completely with a previous whole genome analysis study on *C. auris* [32].

The current initial standard treatments for *C. auris* infection include echinocandins and azoles. Amphotericin B is a second-line agent due to its multiple side effects [28] and recorded high MICs. All patients in our study had high fluconazole MICs and received antifungal treatment with echinocandins (caspofungin or anidulafungin according to the availability of the drug) with a minimum duration of 14 days from the first negative blood culture, except for one patient who was an expatriate and was admitted due to an emergency medical condition while in transit in Oman. He acquired candidemia two days before his transfer back to his country. Antifungals could be given only for two days at the Royal Hospital, but most likely the 14 days were completed in his country of origin. In addition, some patients received combination treatment with liposomal amphotericin B after failing to respond to echinocandins. In addition, CVCs were always removed as a source control measure, and patients kept in isolation room with contact precautions.

We conclude that most *C. auris* infections in this study were seen in critically ill adult patients in the ICU. Patients’ risk factors and antifungal susceptibilities of our isolates were similar to reported cases in the literature. The most common risk factors involved were the presence of CVC and prolonged use of broad spectrum antibiotics. Echinocandins are the empirical drug of choice for *C. auris* infections. Gaps in risk assessment for *C. auris* were not included in the institutional screening protocol during this period, which might have caused epidemiological links in our clinical setting to be missed. A proper screening protocol and risk assessment policy will strengthen infection control measures to prevent *C. auris* infection from becoming a major problem in the future [40,41].

## 4. Materials and Methods

### 4.1. Samples

The clinical records of all patients with positive blood cultures for *C. auris* at the Royal Hospital (Muscat, Oman) in the period from January 2016 to December 2019 were reviewed retrospectively. This hospital is a 630-bed tertiary-care institution. The Hospital provides facilities through the divisions of medicine, surgery, child health, obstetrics, oncology and gynecology. The following critical care wards with 8 beds each are present: the Neonatal ICU, the Pediatric ICU, the Adult ICU, the Coronary Care Unit, and the Post Cardiac Surgery Unit. For this study, blood cultures were taken at the request of the attending physicians, who made the decisions concerning the patients’ diagnosis and treatment. Two to three blood culture sets were usually drawn from an antecubital vein. Blood samples were inoculated into both aerobic and anaerobic blood culture bottles and incubated in the BD BACTECTM FX system (BD, Becton Dickinson) for a maximum of 5 days. Positive bottles were inoculated onto aerobic and anaerobic blood agar (BD Columbia Agar 5% Sheep Blood, Becton Dickinson, Franklin Lakes, NJ, USA), incubating the plates at 35–37 °C for a maximum of 5 days. Anaerobic plates were incubated in an anaerobic atmosphere generated with the AnaeroGen Compact anaerobic system (Oxoid Ltd., Wide Road, Basingstoke, UK) at 35–37 °C. In addition, data collected for each case included demographics, underlying conditions, immunosuppressive medications, clinical signs and symptoms, sites of infection, results from diagnostic tests, pathogen identification, antifungal treatments and outcome. Fungal species identification was performed using phenotypic, genotypic, and proteomic methods.

### 4.2. Laboratory Investigations

Aliquots from positive BACTEC blood culture bottles were Gram stained, and when yeasts were visible, they were subcultured on Sabouraud dextrose agar (SDA) and incubated at 37 °C for 48 h. Yeasts were identified by phenotypic (germ tube formation) and biochemical (VITEK YBC card) tests. Further identification of all isolates was carried out using MALDI-TOF MS (Bruker Biotyper, Bruker Daltoniks, Bremen, Germany), following the manufacturer’s recommendations.

### 4.3. Molecular Identification Using ITS-Sequencing and STR-Genotyping

All isolates (n = 23) we also identified by ITS rRNA gene sequencing using the following primers (ITS1; TCCGTAGGTGAACCTGCGG and ITS4; TCCTCCGCTTATTGATATGC). Strains were transferred to fresh glucose–yeast–peptone agar (GYPA) plates and incubated at 25 °C for 48 h. DNA extraction was performed by the Quick CTAB (cetyltrimethylammonium bromide) extraction, PCR and sequencing methods according to the protocol described by Al-Maani et al. [12].

For genotyping *C. auris,* microsatellite analysis was employed using the following reference strains of clade I (India: ARbank 389), clade II (Japan: ARbank 381), clade III (South Africa: ARbank 383), clade IV (Venezuela: B11245, B11247, B11243, B11244, B1124) and clade V (Iran: ARbank 1097 = IFRC 2087) [39]. Briefly, DNA was extracted and purified with the MagNA Pure LC instrument and the MagNA Pure DNA isolation kit III (Roche Diagnostics GmbH, Mannheim, Germany), according to the recommendations of the manufacturer. Strains were re-suspended in 50 μL physiological salt, and after addition of 200 U of lyticase (Sigma-Aldrich, St. Louis, MO, USA) and incubation for 5 min at 37 °C, 450 μL of physiological salt was added. The sample was then incubated for 15 min at 100 °C and cooled down to room temperature. Four multiplex PCR reactions, which amplify 12 STR targets with a repeat size of 2, 3, or 9 nucleotides, were performed for genotyping the Omani isolates, which were compared with a global collection [39]. Copy numbers of the twelve markers of all isolates were determined using GeneMapper Software 5 (Applied Biosystems). Relatedness between isolates was analyzed using BioNumerics v. 7.6.1 software (Applied Maths, Kortrijk, Belgium) via the unweighted pair group method with arithmetic averages, using the multistate categorical similarity coefficient.

### 4.4. Antifungal Susceptibility Testing

Antimicrobial susceptibility testing of isolates was performed using the M38-A2 broth microdilution method of the CLSI (Clinical and Laboratory Standards Institute) [42].

### 4.5. Ethical Statement

The study did not require oversight by the institutional ethics committee because the descriptive nature implied only samples that were obtained during routine laboratory activity.

## Figures and Tables

**Table 1:** Demographic data of patients, identification and antifungal susceptibility results.

Sample No	MALDI-TOF/Sequencing	Age/Year	Sex	Duration of Treatment (Days)	Time of Candidemia Post Admission (Weeks)	MIC Values (mg/L)							
						AMB	FLC	ITC	VOR	POS	ISA	ANI	MICA
317062126	*C. auris*	54	M	16	3	1	>64	0.125	0.5	0.063	0.063	0.125	0.125
318005583	*C. auris*	50	M	28	2	1	>64	0.125	0.5	0.063	0.031	0.5	0.25
317078724	*C. auris*	78	F	24	12	2	32	0.25	1	0.016	0.5	0.25	0.25
317052804	*C. auris*	69	M	14	1	0.5	64	0.063	0.25	0.016	0.125	0.25	0.125
318014168	*C. auris*	63	F	14	8	1	32	0.063	0.5	0.063	0.5	0.125	0.25
318014517	*C. auris*	30	M	4	NA	1	64	0.031	1	0.031	0.063	0.5	0.25
318028905	*C. auris*	76	F	10	16	1	>64	0.125	1	0.063	0.031	0.25	0.25
318043084	*C. auris*	2	M	16	1	1	>64	0.125	0.5	0.063	0.5	0.125	0.125
318066628	*C. auris*	0.5	M	15	4	2	64	0.5	0.5	0.031	0.063	0.5	0.25
318071689	*C. auris*	83	M	NA	NA	0.5	32	0.25	1	0.125	0.031	0.5	0.125
318095154	*C. auris*	70	F	17	3	1	64	0.125	1	0.063	0.063	0.125	0.25
319008550	*C. auris*	54	M	30	9	2	>64	0.25	0.5	0.063	0.125	0.25	0.125
319056035	*C. auris*	62	M	2	2	2	>64	0.063	0.5	0.063	0.25	0.125	0.125
320000205	*C. auris*	51	M	14	3	1	32	0.031	0.5	0.016	0.25	0.5	0.25
319087097	*C. auris*	62	M	2	5	1	64	0.125	1	0.031	0.063	0.5	0.25
319025492	*C. auris*	50	M	48	15	0.5	>64	0.125	0.5	0.016	0.016	0.5	0.125
319033618	*C. auris*	47	F	16	2	1	32	0.125	0.25	0.016	0.016	0.5	0.25
319023375	*C. auris*	62	M	14	15	2	>64	0.25	0.5	0.063	0.125	0.25	0.25
319089395	*C. auris*	49	F	11	5	1	32	0.063	0.5	0.016	0.125	0.5	0.25
319091222	*C. auris*	54	M	14	6	1	64	0.063	1	0.25	0.031	0.25	0.125
320005972	*C. auris*	31	M	14	1	1	>64	0.5	1	0.125	0.5	0.25	0.125
319094383	*C. auris*	47	M	14	2	1	64	0.125	1	0.031	0.031	0.25	0.125
319029987	*C. auris*	64	F	17	2	1	64	0.125	1	0.25	0.031	0.25	0.125
